# Motivational factors for choosing treatment destinations among the patients treated overseas from the United Arab Emirates: results from the knowledge, attitudes and perceptions survey 2012

**DOI:** 10.1186/s40794-019-0093-9

**Published:** 2019-09-18

**Authors:** Wafa K. Alnakhi, Jodi B. Segal, Kevin D. Frick, Saifuddin Ahmed, Laura Morlock

**Affiliations:** 1 Department of Health Policy and Management Bloomberg School of Public Health, Baltimore, USA; 2 School of Medicine Johns Hopkins University, Baltimore, USA; 3 Carey Business School Johns Hopkins University, Baltimore, USA; 4 Population, Family and Reproductive Health, Baltimore, USA

**Keywords:** Medical travel motivation, Push and pull factors, Treatment destination, Overseas treatment, Medical travel, Travel medicine, United Arab Emirates

## Abstract

**Background:**

Travelling seeking healthcare is becoming common phenomenon. There is limited research to understand factors associated with destinations of choice. Each year the Dubai Health Authority (DHA) spends millions of dollars to cover Emiratis seeking healthcare overseas. The objective of this study is to examine the association of treatment destinations, patients’ characteristics and motivation factors among the patients treated overseas from the UAE during 2009–2012.

**Method:**

The data from the Knowledge, Attitudes and Perceptions Survey 2012 in Dubai on medical travel. Examining destinations by patients’ characteristics and motivational factors under push and pull factor framework. Modified Poisson regression model was used to identify factors associated with treatment destinations.

**Results:**

Three hundred thirty-six UAE national families with a member who sought overseas treatment during 2009–2012 were analyzed for this study regarding their most recent trip. The aim of the survey is to explore their knowledge, attitudes and perceptions. The majority of respondents were family members not the patients who had experienced the medical treatment overseas (63%). Germany was the top treatment destination (45%). The top 3 medical conditions for which people traveled overseas were cancer (17%), bone and joint diseases (16%), and heart diseases (15%). However, patients diagnosed with stroke (brain hemorrhage or clot) are more likely to travel to Germany for medical treatment while patients diagnosed with eye diseases are more likely to seek medical treatment at other destinations. Cost was a primary motivational factor for choosing a treatment destination.

**Conclusion:**

This study addressed knowledge gap related medical travel in the UAE. The results provided evidence about perceptions when choosing treatment destinations. Medical condition and financial factors were main predictors for choosing treatment destination. The result will influence policies related financial coverage by the government. The results suggest understanding patients’ perceptions in-depth related their medical conditions and financial factors for better regulation of overseas treatment strategy in the UAE.

## Introduction

### Background

Dubai is one of the seven Emirates in the United Arab Emirates [1]. The Dubai Health Authority (DHA) is the government entity that oversees the healthcare services in the Emirate of Dubai both as healthcare provider and regulator [2]. By government law, all UAE nationals have free access to healthcare in primary and tertiary healthcare facilities whether they reside or not in Dubai [3]. Although the public healthcare sector strives to provide the best healthcare services to its people, there are still a number of people opt to travel overseas for seeking medical care. The number of patients treated overseas is not accurately enumerated because there are many government entities in the UAE that fund UAE nationals for their treatment overseas. In addition, there are patients who pay out of their pockets. Currently, there is no Emirate or federal level registry that captures the number of medical travelers and their associated expenditure from either the Emirate Dubai or from the other Emirates in the UAE. The average total expenditures per year for overseas treated patients in the Emirate of Dubai according to the DHA from 2004 to 2016 was approximately $77 million US dollars per year for 1500 patients [4]. The most common destinations that patients traveled to are Germany, UK, USA, India, and Thailand. Patients travelling overseas seek an array of treatments from life threatening conditions such as cancer and neurosurgeries to medically optional such as dental and dermatologic procedures [5].

### Study objective

Very limited information was available on the reasons why UAE nationals traveled overseas instead of utilizing healthcare services in Dubai or in other areas of the UAE. Therefore in 2009, the government of Dubai has started to systematically investigate the reasons behind UAE nationals’ travelling overseas for healthcare [6–11]. The government of Dubai is trying to understand the reasons that “pushed” patients from the UAE and “pulled” them towards the treatment destinations [12–17]. The Dubai Health Authority, in collaboration with the Dubai Statistics Center, conducted a knowledge, attitudes and perceptions survey in 2012. Information about overseas treatment is important to both understand the motivational factors underpinning it and help the government to better regulate the medical travel strategy. Furthermore, it will help the government to create evidence base around medical travel related to how people obtain information and make decisions when seeking healthcare overseas.

## Methods

### Data source and study design

This study uses data from a cross-sectional Knowledge, Attitudes and Perceptions (KAP) survey related to medical travel [18]. The data was collected in Dubai, United Arab Emirates between June and July 2012. Total of 361 families UAE nationals and non-UAE nationals who were residents of Dubai with at least one family member experienced seeking healthcare overseas completed the survey. Designing the survey and collecting the data was through a collaborative effort between the Dubai Health Authority (DHA) and the Dubai Statistical Center (DSC). The survey was conducted with nonprobability sampling (purposive sampling) as the methodology of sample selection [19]. The study participants were selected through two main approaches. In the first approach, the sample was drawn from the Dubai Health Authority (DHA) medical records; 452 cases agreed to participate in the survey who had traveled at the government expense during 2010–2012. In the second approach the sample was drawn from the Dubai Statistical Center Household Survey that was conducted in 2009. One hundred nineteen cases agreed to participate in the survey People who had travelled during the same year at their own expense.

### The knowledge, attitudes and perceptions (KAP) survey

The KAP survey was conducted to explore views, perceptions and experiences of UAE residents related to treatment overseas for the period 2009–2012. The survey was developed based on a literature review on medical travel topic. The study was not a validation for a latent scale items. The survey asked the patients (or a family member) about the reasons why the patient travelled overseas. The study aims to understand the motivations behind seeking healthcare abroad instead of seeking healthcare services in the UAE. Both UAE nationals and non-UAE nationals were interviewed who sought healthcare abroad during 2009–2012 [20, 21]. The mode of data collection was through in-person and telephone interviews with times ranging from 45 min to an hour. Patients who were less than 15 years old and patients who were not available for the interview were replaced by a family member 15 years old or above. A family member who either escorted the patient during the treatment abroad and who was eligible to respond to the KAP survey or didn’t escort but had enough information about the patient experience and was eligible to respond. The total number of families who completed the survey was 361 including UAE nationals and non-UAE nationals (63%) out of 571 families with an overseas treated family member identified who was included in the sample. Non-UAE nationals, however, had a low response rate of 22%, so a decision was made to omit them from this analysis and focus only on the 336 UAE nationals who had a response rate of 72%. The decision was made to reduce the noise-effect and focus only on UAE nationals as a priority for this study. The survey included different sections related to patients’ socio-demographics, health seeking behavior in the UAE, travel and treatment experience overseas, family and financial related information, and lastly risks of travel and the satisfaction with medical treatment received overseas. Each item was independently examined with the outcome. Items that are relevant were selected to answer the question as guided by the study conceptual framework and literature review.

### Definition of medical travel

**Fig. 1:**
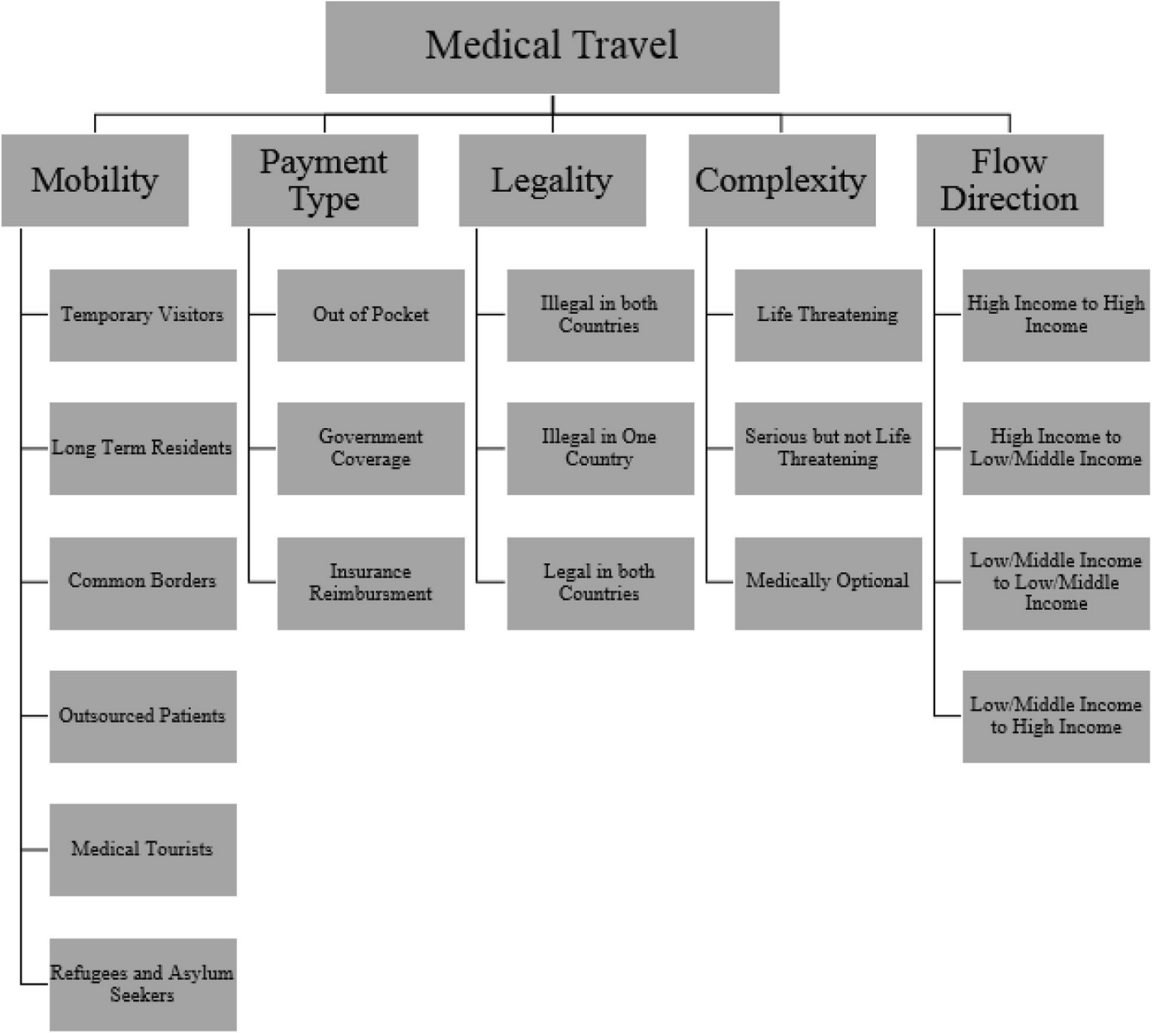
Components of Medical Travel Definition

There is no consensus on the definition of medical travel. All medical travelers are often termed as “medical tourists”, a practice which is not helpful. Therefore, it is important to understand the definition of medical travel; to better understand the motivational factors and estimate their magnitude. According to the literature, there are five main components used to define the medical travel: *1-Patient Mobility 2-Legality 3-Payment Type 4-Complexity Level and 5-Flow Directions* [22, 23]. Medical tourism is defined as the mobility of patients through their own volition and which includes individuals more likely to consider tourism and leisure as a part of the package when seeking medical travel. However, there are other forms of patients’ mobility in which tourism and leisure are not necessary part of the experience [24]. Medical travel defined in this study as the travel of patients from the UAE to treatment destinations for the purpose of legal diagnosis and treatment by UAE law regardless of the level of complexity under the sponsorship of the government or patients paid out of their pocket whether or not tourism and leisure was part of their experience. The shipment of laboratory samples or clinical results for diagnosis and clinical consultations as second options were excluded from the definition of medical travel. This study is limited to patients who travelled in the period of 2009–2012 [25]. Components of medical travel are illustrated in Fig. 1 Appendix A [26–28].

### Conceptual framework

The motivation to travel seeking healthcare has been investigated by researchers in the fields of sociology, anthropology and psychology. Some studies have emphasized that the medical traveler experience can be divided into three chronological stages of the medical travel experience: pre-travel, during travel and post-travel. Understanding each of these stages is necessary to help meet medical travelers’ needs at each stage [29]. Many theories, frameworks, and models are used to explain medical travel. In addition, several studies have sought to understand people’s perceptions when choosing destinations and facilities. Word of mouth, physician recommendation, waiting time, cost of treatment, quality of care, and availability of treatment in the home country appear to be most important when making decisions related to medical travel [30–33].

**Fig. 2:**
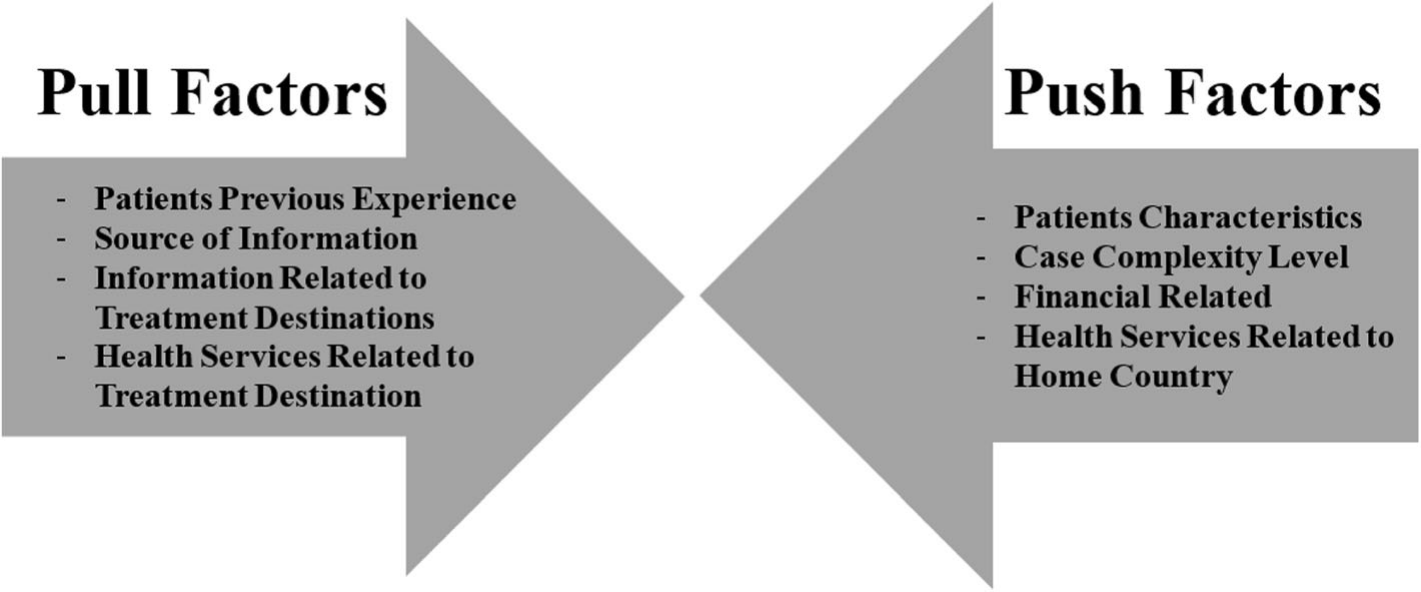
Push and Pull Motivational Factors Conceptual Framework

There are many factors involved in the decision-making process for the medical traveler choice of treatment destinations. Therefore, the Push and Pull Motivational Factors Conceptual Framework may be the most applicable in understanding the motivational factors of the medical travelers in this study. This framework can be viewed as an umbrella that covers most factors related to the medical traveler’s home country and the treatment destinations. Understanding the motivational factors influencing patients treated overseas is important for the health planners, policy makers and governments. This will help them focus their work on rectifying the factors that pushed the patient away from the home country.“The push factors” are defined as the factors that pushed the patients to choose overseas treatment destinations instead of having their treatment in the home country. On the other hand, “the pull factors” are the factors that attracted patients to treatment destination as they are perceived by the patients themselves, as shown in Fig. 2 Appendix B [34, 35].

### Ethical issues

The study protocol was submitted to the Johns Hopkins School of Public Health Institutional Review Board where it was accepted and defined as not human subjects’ research (IRB No: 00007896).

### Statistical analysis

The statistical analyses were conducted by using Stata 13 (Stata Corporation, College Station TX). Quality assurance and quality control of the dataset were performed by running summary statistics for missingness and inconsistencies in the dataset. Means, standard deviations (SD), and student t-tests were used for continuous variables. Percentages, and chi-square tests were used for binary and categorical variables. The outcome was defined as the country destination that residents of Dubai travelled to during the most recent trip before the KAP survey interview. The outcome was dichotomized to travelling to Germany vs other destinations. A modified Poisson regression model was constructed with 95% confidence intervals (CI) and *p* < 0.05 indicating statistical significance since the incidence of traveling to Germany vs other destinations was more than 10% [36]. The Modified Poisson regression model was used to estimate the prevalence ratios (PR) to identify factors associated with treatment destinations. The model was progressively adjusted for different sets of potential confounders. The backward selection method was used to remove variables not statistically significant from the final model. Covariates of two medical conditions (eye disease and stroke), cost of treatment, and treatment coverage were found to be significant. The Akaike information criterion (AIC) test was performed to choose the best fitted model. The model with the lowest AIC was selected indicating the best fit model. The independent variables selected for the model were statistically significant in the bivariate analysis and based on the push-pull factor conceptual framework relevant to our outcome of interest and research question. The variance inflation factor (VIF) was performed to ensure that there is no collinearity among the variables in the final model. The mean VIF was less than 2 which indicated there was no collinearity.

## Results

### Demographic characteristics and treatment destinations

There were 336 UAE national families with a member who sought overseas treatment during 2009–2012 completed the survey regarding their most recent trip to explore their knowledge, attitudes and perceptions. The majority of survey respondents were eligible family members (63%). Those family members either escorted patients during the overseas treatment experience or didn’t escort the patient but did have enough information about the patient experience to serve as survey respondents. The patients treated overseas travelled to 17 destinations. The top destinations for treatment overseas among residents of Dubai were: Germany (45%), followed by Thailand (19%), and UK (11%). Other less frequent destinations are shown in Table 1. The gender of the overseas treated patients was equally distributed among males and females. The patients’ mean age was 40.09 ± 22.66; a higher proportion were married (66%), not working (66%), with up to a high school education (49%), and lower household income (60%). Patients who travelled to Germany were more likely than those travelling to other destinations to have mid-level or higher household incomes (*P* = 0.045), as shown in Table 2.

**Table 1:** Top treatment destinations of residents of Dubai, United Arab Emirates who sought medical treatment overseas during 2009–2012

No.	Treatment Destination	Total Sample N (%)
1	Federal Republic of Germany	152 (45)
2	Kingdom of Thailand	64 (19)
3	United Kingdom	37 (11)
4	Republic of India	27 (8)
5	United States of America	13 (4)
6	Republic of Singapore	13 (4)
7	Kingdom of Belgium	8 (2)
8	Republic of Austria	5 (1)
9	Other destinations^a^	17 (5)
	Total	336 (100)

^a^Other destinations: Hashemite Kingdom of Jordan, Ireland, Islamic Republic of Iran, French Republic, Islamic Republic of Afghanistan, Republic of Indonesia, Kingdom of Spain, Other Asian countries, Other Latin America countries

**Table 2:** Demographic characteristics of residents of Dubai, United Arab Emirates who sought medical treatment overseas during 2009–2012

Variable	Total Sample N (%)	Federal Republic of Germany N (%)	Other Destinations N (%)	*P* -value
Gender				1.00
Male	168 (50)	76 (50)	92 (50)	
Female	168 (50)	76 (50)	92 (50)	
Age (years) ^a^	40.09 ± 22.66	38.90 ± 22.91	41.08 ± 22.46	0.38
Marital Status^b^				0.56
Married	177 (66)	75 (64)	102 (68)	
Not Married	91 (34)	42 (36)	49 (32)	
Employment Status^b^				0.85
Not working	178 (66)	77 (66)	101 (67)	
Working	90 (34)	40 (34)	50 (33)	
Educational Level^b^				0.89
Illiterate or Can’t Read & Write	73 (27)	32 (27)	41 (27)	
Up to High School	132 (49)	56 (48)	76 (50)	
College & Above	63 (24)	29 (25)	34 (23)	
Household Income^c^				0.045
Low Income	203 (60)	81 (53)	122 (66)	
Middle Income	70 (21)	39 (26)	31 (17)	
Higher Income	63 (19)	32 (21)	31 (17)	
Answering the Survey				0.21
Self-reported	125 (37)	51 (34)	74 (40)	
Family member reported	211 (63)	101 (66)	110 (60)	
Family Member Reported				0.92
Escorted	189 (92)	92 (92)	97 (92)	
Not Escorted	16 (8)	8 (8)	8 (8)	

^a^ mean ± standard deviation

^b^ Only among those who are 15 years and older

^c^1 AED = 0.272294 USD / 1 USD = 3.67250 AED [low income (≤29,000 AED = ≤7,986.53 USD), middle income (≥30,000 - ≤99,999 AED = ≥8,168.82 - ≤27,229.14 USD), high income (≥100,000 AED = ≥ 27,229.41 USD)] currency rate in 2018

### Health seeking behavior before travelling overseas

Before seeking medical treatment overseas (82%) of patients were diagnosed for their medical conditions in the UAE. The majority of patients did consult their healthcare providers before travelling overseas (85%). There were (64%) of patients who sought medical treatment overseas and who received healthcare services in the government/public sector for their healthcare conditions before obtaining medical treatment overseas^1^. Overall, patients who traveled overseas either to Germany or other destinations had a mean satisfaction rating of 1.88 ± 1.34 which indicates they were neither satisfied nor dissatisfied with the healthcare services they received in the UAE^2^ as shown in Table 3 and Appendix C.

**Table 3:** Residents of Dubai, United Arab Emirates health seeking behavior before travelling overseas during 2009–2012

Variable	Total Sample N (%)	Federal Republic of Germany N (%)	Other Destinations N (%)	*P*-value
Health Situation				
Undiagnosed	59 (18)	25 (16)	34 (18)	0.63
Diagnosed	277 (82)	127 (84)	150 (82)	
Consult Healthcare Provider				
Didn’t Consult	51 (15)	20 (13)	31 (17)	0.35
Consult	285 (85)	132 (87)	153 (83)	
Healthcare Provider				
Government	215 (64)	100 (66)	115 (63)	0.53
Other	121 (36)	52 (34)	69 (38)	
Satisfaction of the Healthcare Services Provided in the UAE				1.00
Very dissatisfied	72 (21)	32 (21)	40 (22)	
Dissatisfied	69 (21)	31 (20)	38 (21)	
Neutral	60 (18)	27 (18)	33 (18)	
Satisfied	96 (29)	45 (30)	51 (28)	
Very Satisfied	39 (12)	17 (11)	22 (12)	
Mean ± SD^a^	1.88 ± 1.34	1.89 ± 1.34	1.88 ± 1.35	0.89

^a^ mean ± standard deviation

### Diagnosis and medical condition at baseline before seeking healthcare overseas

The top 3 medical conditions for which people traveled overseas were cancer (17%), bone and joint diseases (16%), and heart diseases (15%). Patients who travelled to other destinations were more likely to have been diagnosed with eye diseases (*p* = 0.01), while patients who traveled to Germany were more likely to have been diagnosed with stroke (brain hemorrhage or clot) (*p* = 0.03) as shown in Table 4.

**Table 4:** Main conditions residents of Dubai, United Arab Emirates were diagnosed with before seeking medical treatment overseas during 2009–2012^a^

No.	Medical Condition	Total Sample N (%)	Federal Republic of Germany N (%)	Other Destinations N (%)	*P*-value
1	Cancer	47 (17)	21 (17)	26 (17)	0.84
2	Bone and Joint	44 (16)	23 (18)	21 (14)	0.35
3	Heart Diseases	41 (15)	17 (13)	24 (16)	0.54
4	High Blood Pressure	24 (7)	7 (5)	17 (9)	0.10
5	Diabetes	34 (10)	12 (8)	22 (12)	0.22
6	Gastroenterology	22 (7)	10 (7)	12 (7)	0.98
7	Eye Disease	20 (6)	3 (2)	17 (9)	0.01
8	Urinary System	16 (5)	9 (6)	7 (4)	0.36
9	Obstetrics and Gynecology	8 (2)	1 (1)	7 (4)	0.06
10	Lungs and Respiratory	9 (2)	3 (2)	6 (3)	0.47
11	Trauma	8 (2)	3 (2)	5 (3)	0.66
12	Stroke	7 (2)	6 (4)	1 (1)	0.03
13	Ear, nose and throat (ENT) Diseases	3 (1)	2 (1)	1 (1)	0.45
14	Cosmetic	3 (1)	2 (1)	1 (1)	0.45
15	Skin and Venereal Diseases	2 (1)	1 (1)	1 (1)	0.90
16	Oral and Dental Diseases	1 (< 1)	0 (0)	1 (1)	0.36

^a^More than one choice for medical condition was permitted

### Motivational factors for seeking medical treatment overseas

Almost all of the patients (99%) who travelled overseas indicated that they went for treatment only and not for other purposes^3^. Overall, those patients who travelled overseas either to Germany or to other destinations had no differences regarding their motivational factors ^4^, including: having a previous experience in the treatment destination, importance of vacation aspects, a country has a friendly environment, and following someone’s advice. On the other hand; patients who travelled to Germany were less likely than those travelled to other destinations to cite the cost of travel as an important factor in their decision-making for seeking healthcare overseas, (*p* = 0.002). When respondents were asked about sources of information utilized, (54%) reported using a physician’s recommendation as a source of information when travelling overseas, followed by word of mouth from family and friends (52%). Moreover, (28%) of respondents reported they would look at the physician’s experience first when choosing a healthcare provider for services overseas. The majority of respondents (76%) stated that they inquired about the physician at the treatment destination; in addition, (57%) inquired about the physician’s training and qualifications. Patients who sought treatment in Germany were less likely to ask about the costs of treatment and follow-up than patients who travelled to other destinations (*p* = 0.01) when inquiring about the physician overseas. When asked about their main reason for travelling overseas, (9%) of patients stated that long waiting time for an appointment was the main reason for deciding to obtain healthcare services overseas, as shown in Table 5 and Appendix C.

**Table 5:** Motivational factors among residents of Dubai, United Arab Emirates who sought medical treatment overseas during 2009–2012

Variable	Total Sample N (%)	Federal Republic of Germany N (%)	Other Destinations N (%)	*P*-value
Main Reason for Travel				0.85
Treatment purpose only	332 (99)	150 (99)	182 (99)	
Other purposes	4 (1)	2 (1)	2 (1)	
Have Been There Before				0.19
Not Important at all	166 (49)	74 (49)	92 (50)	
Not Important	56 (17)	33 (22)	23 (13)	
Neutral	13 (4)	6 (4)	7 (4)	
Important	58 (17)	23 (15)	35 (19)	
Very Important	43 (13)	16 (11)	27 (15)	
mean ± SD^a^	2.27 ± 1.52	2.17 ± 1.43	2.35 ± 1.58	
Vacation Aspects				0.11
Not Important at all	218 (65)	100 (66)	118 (64)	
Not Important	66 (20)	36 (24)	30 (16)	
Neutral	25 (7)	9 (6)	16 (9)	
Important	18 (5)	4 (3)	14 (8)	
Very Important	9 (3)	3 (2)	6 (3)	
mean ± SD^a^	1.61 ± 1.01	1.51 ± 0.87	1.70 ± 1.11	
Friendly Atmosphere				0.24
Not Important at all	145 (43)	71 (47)	74 (40)	
Not Important	47 (14)	25 (16)	22 (12)	
Neutral	22 (7)	10 (7)	12 (7)	
Important	68 (20)	28 (18)	40 (22)	
Very Important	54 (16)	18 (12)	36 (20)	
mean ± SD^a^	2.52 ± 1.58	2.32 ± 1.50	2.68 ± 1.63	
Advised by Someone				0.53
Not Important at all	62 (18)	31 (20)	31 (17)	
Not Important	30 (9)	16 (11)	14 (8)	
Neutral	22 (7)	12 (8)	10 (5)	
Important	81 (24)	33 (22)	48 (26)	
Very Important	141 (42)	60 (39)	81 (44)	
mean ± SD^a^	3.62 ± 1.54	3.49 ± 1.58	3.73 ± 1.50	
Cost of Treatment				0.002
Not Important at all	155 (46)	81 (53)	74 (40)	
Not Important	54 (16)	29 (19)	25 (14)	
Neutral	41 (12)	17 (11)	24 (13)	
Important	32 (10)	13 (9)	19 (10)	
Very Important	54 (16)	12 (8)	42 (23)	
mean ± SD^a^	2.33 ± 1.51	1.99 ± 1.31	2.612 ± 1.62	
Sources of Information Used to Travel Abroad				
Word of mouth family and friends	176 (52)	74 (49)	102 (55)	0.22
Internet forums	61 (18)	30 (20)	31 (17)	0.49
Magazine/newspaper	1 (< 1)	1 (1)	0 (0)	0.27
Radio/TV	1 (< 1)	0 (0)	1 (1)	0.36
Brochures and leaflets	1 (< 1)	1 (1)	0 (0)	0.27
Literature	2 (1)	1 (1)	1 (1)	0.89
Physician’s recommendations	181 (54)	88 (58)	93 (51)	0.18
Providers webpage	4 (1)	2 (1)	2 (1)	0.85
Medical Travel agency/Broker	2 (1)	1 (1)	1 (1)	0.89
Government (overseas treatment office)	80 (24)	41 (27)	39 (21)	0.22
Information Would Use to Choose Healthcare Provider				0.41
Different Treatment Options	27 (8)	14 (9)	13 (7)	
Qualifications and certificates of the doctor	39 (12)	19 (13)	20 (11)	
Experience of the doctor	95 (28)	36 (24)	59 (32)	
Reputation of the medical center/hospital	80 (24)	36 (24)	44 (24)	
Past success stories	41 (12)	19 (13)	22 (12)	
Cost of treatment	6 (2)	2 (1)	4 (2)	
Cost of accommodation, air fare, transport, food, etc.	1 (< 1)	0 (0)	1 (1)	
Length of stay	1 (< 1)	1 (1)	0 (0)	
Adverse outcomes and complications of the desired treatment	2 (1)	2 (1)	0 (0)	
Refund policy	2 (1)	0 (0)	2 (1)	
The probability of having the treating doctor abroad as visiting doctors in the UAE for consultations	10 (3)	7 (5)	3 (2)	
Available advanced medical & Therapeutic technology	3 (1)	1 (1)	2 (1)	
Opinions of friends and family regarding the best healthcare providers in the city/country	29 (9)	15 (10)	14 (8)	
Inquire About Physician				0.76
Didn’t Inquire	80 (24)	35 (23)	45 (24)	
Inquire	256 (76)	117 (77)	139 (76)	
Types of Inquiries About the Physician Abroad				
Physician Training & Qualifications	191 (57)	86 (57)	105 (57)	0.93
Recovery Time as inpatient	128 (38)	55 (36)	73 (40)	0.51
How soon will travel back home	87 (26)	37 (24)	50 (27)	0.56
Pictures of Previous Patients	59 (18)	29 (19)	30 (16)	0.51
Complications & Adverse outcomes	84 (25)	38 (25)	46 (25)	1.00
Cost of treatment and follow up	35 (10)	9 (6)	26 (14)	0.01
Main reason to travel overseas for Healthcare				
Cannot afford treatment in the UAE	12 (4)	6 (4)	6 (3)	0.74
Not eligible for the service provided in the UAE	11 (3)	5 (3)	6 (3)	0.99
Long waiting time for an appointment	29 (9)	12 (8)	17 (9)	0.66
Undesirable outcome from previous personal experience	21 (6)	8 (5)	13 (7)	0.50
Undesirable outcome from other previous experience	24 (7)	8 (5)	16 (9)	0.22
Privacy and confidently reasons	27 (8)	10 (7)	17 (9)	0.37
Healthcare provider attitude	20 (6)	5 (3)	15 (8)	0.06
Post treatment rehabilitation is not available	6 (2)	3 (2)	3 (2)	0.81
Expecting adverse treatment outcome in the UAE	20 (6)	7 (5)	13 (7)	0.34

^a^ mean ± standard deviation

### Travel related experience

The average number of months was 15.66 ± 15.71 from the most recent trip to being interviewed for the KAP survey. Overall, (68%) of patients who received medical services overseas had inpatient treatment^5^, with patients traveling to Germany more likely to receive inpatient services than those travelling to other destinations (*p* = 0.042). More than half of the patients who sought healthcare services overseas (56%) stated that their medical treatment was not available in the UAE. Overall, the majority (79%) indicated that their expenses of treatment were covered by the government. Those who travelled to Germany were significantly more likely to have government coverage than those travelling to other destinations^6^ (*p* = < 0.001). The majority of the respondents (88%) revealed that they didn’t know about the refund policy of the health care provider overseas. Overall, patients who received overseas medical treatment had a higher mean satisfaction level 3.45 ± 0.94 with the healthcare received during the last trip overseas than with the healthcare services they had received in the UAE. The great majority of respondents (90%) would recommend their overseas healthcare trip experience to someone else. When survey participants were asked about the aspects of services they would like to have available in the UAE, the top 3 were: good healthcare provider communication^7^ (82%), convenient access and atmosphere^8^ (64%), and a reasonable waiting time at the clinic^9^ (42%).

Although most patients (82%) who received medical treatment overseas did not experience any unfavorable reactions/complications/outcomes during or after treatment overseas, patients who travelled to Germany were more likely to experience such events^10^ (*p* = 0.002). The majority (85%) of the respondents expressed that they knew where to report in case of a medical error and (71%) indicated they would contact the UAE embassy at the destination country. In addition, the majority of respondents (76%) expressed that they would wait and still go to the same destination if they faced a delay in the issuing of a visa of entry to their desired destination^11^ as shown in Table 6 and Appendix C [37].

**Table 6:** Travel related experience for residents of Dubai, United Arab Emirates who sought medical treatment overseas during 2009–2012

Variable	Total Sample N (%)	Federal Republic of Germany N (%)	Other Destinations N (%)	*P*-value
Months ago was the trip Mean ± SD^a^	15.66 ± 15.71	16.53 ± 15.26	14.93 ± 16.09	0.37
Type of Healthcare Services				0.042
Inpatient	228 (68)	113 (74)	115 (61)	
Outpatient	102 (30)	38 (25)	64 (35)	
Unknown	6 (2)	1 (1)	5 (3)	
Treatment Available in the UAE				0.08
Available	96 (29)	40 (26)	56 (30)	
Not Available	187 (56)	94 (62)	93 (51)	
I don’t know	53 (16)	18 (12)	35 (19)	
Treatment Coverage				< 0.001
Government Expenses	265 (79)	141 (93)	124 (67)	
Other Sources	71 (21)	11 (7)	60 (33)	
Refund Policy Healthcare Abroad				0.71
I know	40 (12)	17 (11)	23 (13)	
I don’t know	296 (88)	135 (89)	161 (88)	
Satisfaction of the Healthcare Services Provided Overseas				0.06
Very dissatisfied	12 (4)	7 (5)	5 (3)	
Dissatisfied	8 (2)	7 (5)	1 (1)	
Neutral	12 (4)	6 (4)	6 (3)	
Satisfied	89 (26)	44 (29)	45 (24)	
Very Satisfied	215 (64)	88 (58)	127 (69)	
Mean ± SD	3.45 ± 0.94	3.31 ± 1.06	3.56 ± 0.82	
Recommending Overseas Experience to Others				0.99
Recommend	302 (90)	137 (90)	165 (90)	
Don’t Recommend	33 (10)	15 (10)	18 (10)	
Aspects of Services Wish to Be Available in the UAE				0.11
Waiting time	142 (42)	57 (38)	85 (46)	0.11
Healthcare provider Communication	277 (82)	121 (80)	156 (85)	0.21
Hospitality	89 (26)	45 (30)	44 (24)	0.24
Education & Reading Material	17 (5)	10 (7)	7 (4)	0.25
Convenient Atmosphere	215 (64)	92 (61)	123 (67)	0.23
Unfavorable Reactions/ Complications/ Outcomes During and After the Treatment				0.002
No	274 (82)	113 (74)	161 (88)	
Yes	62 (18)	39 (26)	23 (13)	
I know where to report medical error				0.16
I don’t Know	50 (15)	18 (12)	32 (17)	
I know	286 (85)	134 (88)	152 (83)	
Where to Report medical error				
Embassy	237 (71)	109 (72)	128 (70)	0.67
Overseas Patients Affairs Office	95 (28)	47 (31)	48 (26)	0.33
Police	15 (4)	6 (34)	9 (5)	0.68
Hospital Administration /complaint center	40 (12)	18 (12)	22 (12)	0.97
Next decision if there was delay in issuing visa				0.19
Wait for Visa	257 (76)	119 (78)	138 (75)	
Look for Another Destination	54 (16)	26 (17)	28 (15)	
Search HCP in UAE^12^	25 (7)	7 (5)	18 (10)	

^a^ *SD* Standard Deviation

With regard to preferences for travelling overseas for treatment and the role of family members, the great majority of the respondents (97%) preferred travelling overseas escorted by a family member and trip to be arranged by a travel agency (72%). The majority of respondents disclosed that their family’s response was to support and help their decision about travelling overseas for medical treatment (93%). However, financial help from the family was less likely for those travelling to Germany in comparison to those who travelled to other destinations (*p* = 0.02) as shown in Table 7.

**Table 7:** Preferences and family related questions for residents of Dubai, United Arab Emirates United Arab Emirates who sought medical treatment overseas during 2009–2012

Variable	Total Sample N (%)	Federal Republic of Germany N (%)	Other Destinations N (%)	*P*-value
Preference for Travel Escort				0.76
Alone	10 (3)	5 (3)	5 (3)	
Escorted	326 (97)	147 (97)	179 (97)	
Arrangement Preferences				0.81
Myself	95 (28)	42 (28)	53 (29)	
Travel Agency	241 (72)	110 (72)	131 (71)	
Family response towards overseas treatment				
Shared bad experiences	29 (8)	14 (9)	15 (8)	0.73
Help & Support	314 (93)	143 (94)	171 (93)	0.67
Seek Options in UAE/Other Countries	39 (12)	20 (13)	19 (10)	0.42
Financial Help	87 (26)	30 (20)	57 (31)	0.02
Worry	47 (14)	19 (13)	28 (15)	0.48

### The motivational factors and associations with treatment destinations when seeking healthcare services overseas

Unadjusted and adjusted prevalence ratios are shown in Table 8. People diagnosed with eye diseases had a 66% lower prevalence ratio of choosing Germany as a destination of treatment compared to people with other medical conditions (PR 0.34, 95% CI: 0.13, 0.87). On the other hand, people who were diagnosed with stroke (brain hemorrhage or clot) had a 90% higher prevalence ratio to choose Germany compared to people with other medical conditions as a destination of treatment (PR 1.90, 95% CI: 1.45,2.51). People who had the cost of treatment as an important factor when choosing treatment destination had a 29% lower prevalence ratio of choosing Germany compared to people who reported cost as not important at all (PR 0.71, 95% CI: 0.51,0.10). People who were not sponsored by the government had a 67% lower prevalence ratio of choosing Germany as a treatment destination compared to people who were sponsored by the government (PR 0.33, 95% CI: 0.19).

**Table 8:** Unadjusted and adjusted prevalence ratios for travelling to Germany compared to other treatment destinations among residents of Dubai, United Arab Emirates during 2009–2012

Dependent Variables	Unadjusted			Adjusted^a^		
	PR	95% CI	*P*-value	PR	95% CI	*P*-value
Medical Condition						
Other Diseases	1.00	–	–	1.00	–	–
Eye Diseases	0.31	(0.11,0.91)	0.03	0.34	(0.13,0.870)	0.03
Other Diseases	1.00	_	_	1.00	_	_
Stroke (brain hemorrhage or clot)	1.93	(1.40,2.68)	< 0.001	1.90	(1.45,2.51)	< 0.001
Cost of Treatment						
Cost is not Important at all	1.00	–	–	1.00	–	–
Indifferent about the cost	0.79	(0.54,1.16)	0.23	0.83	(0.57,1.21)	0.34
Cost is very important	0.55	(0.39,0.79)	0.001	0.71	(0.51,1.00)	0.05
Treatment Coverage						
Government coverage	1.00	–	–	1.00	–	–
Non-Government coverage	0.29	(0.17,0.51)	< 0.001	0.33	(0.19,0.57)	< 0.001

^a^Adjusted for medical condition, cost of treatment, treatment coverage and using Modified Poisson as a model of Analysis

## Discussion

Nearly half of the patients from the United Arab Emirates who were interviewed for the Knowledge, Attitudes and Perceptions survey travelled to Germany as a treatment destination during 2009–2012. The prevalence of travelling to Germany was significantly associated with lower concerns about financial costs and with having government coverage for medical expenses. Patients who travelled to Germany were more likely to be diagnosed with stroke (brain hemorrhage or clot) and less likely to be diagnosed with eye diseases.

Many studies have stated that financial cost plays a vital role in influencing decisions regarding seeking healthcare services overseas [38–47]. Respondents to this survey were price sensitive when making the decision between seeking healthcare in Germany compared to other destinations [48]. Respondents agreed that cost is very important when choosing the destination. Cost was part of respondents concerns when inquiring about physicians abroad. Patients’ families were more likely to provide financial support when patients made the decision to travel to other destinations compared to Germany. In addition, patients travelling to Germany were more likely to have their medical expenses covered by the government compared to other destinations. On the other hand; medical conditions were another factor influencing choice of the country of destination. Patients diagnosed with stroke (brain hemorrhage or clot) were more likely to choose Germany as a treatment destination. On the contrary, patients diagnosed with eye disease were more likely to choose other destinations compared to Germany.

According to the literature, there are many motivational factors that can push the patients from the country of residency and pull them towards the treatment destinations. Although the financial cost was a significant reason for choosing between Germany and other destinations, other factors were also important in seeking healthcare services overseas. Being advised by someone, word of mouth from family and friends, a physician’s recommendation, and long waiting time for treatment in the UAE are all important factors [49, 50]. The literature emphasizes the importance of word of mouth as a source of information when exchanging and looking for feedback about the treatment destination. In addition, the literature has stated that people’s expectations are formed as a result of word of mouth and recommendations. The word of mouth could be either from family and friends or a physician’s referral and recommendations. Quality of care, long waiting time and unavailability of the treatment in the country of residence are considered fundamental factors that push people to treatment destinations. People would prefer destinations that are specialized for the healthcare services related to their health condition. Furthermore, healthcare providers’ interpersonal aspects, conduct and communication, as well as medical staff responsiveness are important factors in seeking healthcare services overseas. In our study respondents expressed that the type of healthcare provider communication experienced overseas would be desirable in the UAE.

Although physician reputation and characteristics were not significant variables in choosing between the different destinations in our study, 76% of the respondents stated that they would inquire about the physician abroad before seeking healthcare services overseas. Some stated they would inquire about physician training and qualifications and others would inquire about recovery time as an inpatient. Physician characteristics are one of the important factors when selecting a healthcare provider overseas [51]. Physician competence, expertise, training and qualifications were selected by our respondents in the survey, which is consistent with the literature [52]. Some studies have demonstrated that physician demographic characteristics such as age, gender, race, religion and marital status are least important to the patients when making a choice about physicians compared to physicians’ professional expertise such as being board certified and specializations. We should be careful about generalizing this information since patients coming from different cultures might differ in their preferences. Hospital reputation, accreditation and characteristics are other important factors, following physician characteristics when selecting healthcare providers [53]. According to some studies about patients’ hospital choices, in non-emergency cases and when patients are financially covered, patients will choose hospitals with high quality of care ratings and shorter waiting times. Furthermore patients’ decisions are more influenced sometimes by family and friends’ experiences when making a decision about a hospital or a medical center compared to the key performance indicators of the healthcare provider on its webpage [54].

Acknowledging limitations of the study is very important in order to make suggestions for future research related to seeking treatment overseas. The sample size was relatively small for this study and many motivational factors that were considered significant in the literature were unable to be detected as significant in this study. Therefore, to achieve a desired level of precision and a desired margin of error, a minimum detectable difference is required through a power and sample size calculation to have a better representative sample in the future [55]. Since the methodology was through purposive sampling, we have to be careful with generalization. The participants in the study are not the true representation of the population of the Emirate of Dubai nor the UAE which would be needed to make statistical inferences. Additionally, the ratio of non-UAE nationals to UAE nationals was 3:47 (6:94) which is not the true representation of the population of Dubai. Therefore, the non-UAE nationals were dropped deliberately from the sample to reduce the “noise effect” and to focus on the UAE-nationals only as a priority in this study. It is also worthwhile to mention that our study is a cross-sectional study; as a result, it yields weak evidence of causality between the predictors and the outcome.

Only 37% of the surveys were answered by the patient who had experienced the medical treatment overseas. This leads to the question of whether the perceptions and the motivational factors identified in this survey reflect the true perceptions of the patients or the family members who answered the survey. In addition, since 16 months was the average time from the most recent trip and being interviewed for this survey, “re-call bias” may pose a threat to the internal validity of the survey results. Moreover, the study design did not account for whether more than one family member experienced travelling overseas for medical treatment. Accounting for more than one family member would help ensure that the survey is capturing the right experiences adjusted for the patient characteristics, treatment destination, motivational factors and medical condition. Validity and reliability of the survey can be further improved in the future. Although the survey was piloted once, it is important to use the survey more than one time on the same population to test the reliability and consistency of the tool overtime [56, 57].

Regarding the strengths of this study, although there is some literature about medical travel, there is very limited quantitative research studying the associations between patients’ characteristics, motivational factors, and medical conditions when choosing treatment destinations, physicians and hospitals in the treatment destinations. Therefore, this research provides good insights and will contribute to the knowledge base regarding seeking healthcare overseas. This study will have great policy and strategy implications, not only for the Emirate of Dubai, but also for the UAE in general.

Understanding the motivational factors for people who traveled overseas seeking healthcare will help in creating strategies to improve the healthcare services in the Emirate of Dubai and in the UAE. Moreover, it will give better insights for having long term planning for better access with alternative options for patients in the government sector and the private sector in the Emirate of Dubai. That strategy can be achieved through the expansion of the healthcare services related to the medical conditions patients travelled for and also through collaboration between the government and private sector through public-private partnership agreements [58, 59].

It is also important for the government to ensure that the patients have adequate information about the services related to their medical condition in the UAE. This will give patients more options to choose from and increase patients’ access and utilization of the healthcare services in Dubai and in the UAE. Identifying the pull and push factors are also important in order to use them to attract patients to stay in the UAE. This will reduce the risks and complications following treatment overseas, since patients will be diagnosed and treated locally instead of having treatment and follow up in two different locations.

Some respondents expressed that long waiting time, as well as privacy and confidentiality reasons, were main motives to travel overseas. Thus, the government should work on reducing waiting time and ensure policies and regulations are in place to protect privacy and patients’ rights. In addition, it is important to underscore that healthcare provider communication was one of the service aspects that respondents wished to be available in the UAE. Therefore physicians, nurses, allied health personal and all the workforce who provide healthcare services or who are in a direct contact with the patients should be trained for better interpersonal communication.

## Conclusion

To conclude, our study contributes towards understanding the motivational factors for choosing treatment destinations in the field of medical travel. Our results have demonstrated that medical condition and financial factors are associated with choosing treatment destinations. It is important to understand medical travelers’ motivational factors to create an evidence base for the government and for the patients when making future decisions related to treatment destinations. Creating evidence base will influence and promote better patients’ informed decisions, will guide the government to improve the quality of care provided in the UAE and will influence policies related financial coverage by the government.

## Data Availability

The data that support the findings of this study are available from the Dubai Health Authority and Dubai Statistics Center, but restrictions apply to the availability of these data, which were used under special agreement for the current study, and so are not publicly available. Data are however available from the corresponding author upon reasonable request and with permission of the Dubai Health Authority and Dubai Statistics Center.

## Appendix A

### Components of Medical Travel Definition

**Mobility:**

- **Temporary visitor:** individuals vacationing overseas and utilizing healthcare services of the country of destination due to sudden illness or accidents
- **Long term residents:** retirement migration
- **Common border:** countries sharing common border may collaborate in providing financial health coverage
- **Refuges and asylum seekers:** individuals who cross borders due to political crisis or instability in their home country
- **Outsourced patients:** patients who are sent overseas by health agencies or authorities thorough cross-national agreement driven by long waiting time, unavailability of the services in the home country, quality of care and cost of treatment

**Payment Type:**

- **Out of pocket:** individual expenses for medical care that are not reimbursed by government or insurance agency
- **Government coverage:** public entity or authority responsible of covering individuals’ healthcare expenses
- **Insurance reimbursement:** an agency that is responsible of reimbursing individuals’ expenses for medical care under certain agreements

**Legality:**

- **Illegal in both countries:** medical treatment is illegal in patients’ home country and treatment destination
- **Illegal in one country:** medical treatment is illegal in patients’ home country but legal in the treatment destination
- **Legal in both countries:** medical treatment is legal in patients’ home country and treatment destination

**Complexity:**

- **Life threatening:** the primary condition can cause immediate death or eventual death in the person unless it is interrupted with a treatment
- **Serious but not life threatening:** the primary condition is not causing immediate or eventual death but may progress to a life threatening if left untreated
- **Medically optional:** services that are not reasonable and necessary for the diagnosis or treatment of illness or injury or to improve the functioning of a malformed body member, it is a life enhancing medical treatment or procedure

**Flow Direction:**

- **High income to high income:** individuals from countries with high gross national income (GNI), seeking health care in countries with similar gross national income (GNI) as per the World Bank country classification
- **High income to low/middle income:** individuals from countries with high gross national income (GNI), seeking healthcare in countries with low/middle gross national income (GNI) as per the World Bank country classification
- **Low/middle income to high income:** individuals from low/middle gross national income (GNI), seeking healthcare in countries with high gross national income (GNI) as per the World Bank country classification
- **Low/middle income to low middle income:** individuals from low/middle gross national income (GNI), seeking healthcare in countries with similar gross national income (GNI) as per the World Bank country classification

## Appendix B

### Push and Pull Motivational Factors Conceptual Framework

**Push Motivational Factors:**

- **Patient’s Characteristics**: patient’s socio-demographics
- **Complexity Level:** life threatening, serious but not life threatening, medically optional
- **Financial Related**: payment type (out of pocket payment, government coverage, private insurance reimbursement)
- **Health Services Related to Home Country:** long waiting time, unavailability of treatment in the home country, quality of care

**Pull Motivational Factors:**

- **Patient Previous Experience:** patient previous experience with quality of care or advanced technology in the treatment destination
- **Source of Information:** scholarly sources, media sources, word of mouth
- **Information Related to Destination Country:** economic and political stability, safety, attractiveness, geographic closeness, health policies and regulations
- **Health Services Related to destination countries:** quality of care, specialists, or advanced technology in medical centers at the treatment destination

## Appendix C

### Knowledge, Attitudes and Perceptions Survey 2012

1. Government/public sector: Dubai Health Authority inpatient/outpatient services, Abu Dhabi Health Services Hospitals and PHCs (SEHA), and Ministry of health inpatient/outpatient services
2. Satisfaction level of the healthcare services provided in the UAE: Likert scale 0 = Very dissatisfied, 1 = Dissatisfied, 2 = Neutral, 3 = Satisfied, 4 = Very Satisfied
3. Travelled overseas for other purposes: tourism or business
4. Motivational factors: Likert scale 0 = Not Important at all, 1 = Not Important, 2 = Neutral, 3 = Important, 4 = Very Important
5. Inpatient treatment: surgical or non-surgical
6. Government coverage: government of Dubai, or Ministry of health, or Government of Abu-Dhabi
7. Healthcare provider communication: treating doctor talked clearly to me about my condition, Treating doctor gave me different treatment options, Treating doctor explained to me how I can cope; live normal life with my condition, Treating doctor explained what might happen to me in the future, The medical staff was polite, and courteous, The medical staff was able to respond to my inquiries efficiently and referred me to the right persons, The treating doctor was listening to me
8. Convenient access and atmosphere: easiness of booking for an appointment “convenient, didn’t take long time”, Consultation and Diagnostic work-ups and treatment were all in the same building, the hospital called me to report my results instead of me going to the hospital
9. Reasonable waiting time at the clinic before seeing the doctor
10. Unfavorable reactions/complications/outcomes during or after treatment overseas: fever/infection after the surgery, allergy from medication, wrong diagnosis, other surgical complications, other medical complications, results not as explain by the doctor
11. Visa: the survey was designed and conducted before the agreement between the European Union and the United Arab Emirates in Brussels on May 6th, 2015 on the short-stay visa waiver were Ireland and the United Kingdom are not part of this agreement
12. HCP: healthcare provider
